# Gut microbial markers of immunotherapy response in melanoma: a cross-cohort analysis including the first Russian dataset

**DOI:** 10.1080/19490976.2026.2681788

**Published:** 2026-06-15

**Authors:** Aleksandra Strokach, Natalia Zakharevich, Viktoriya Aginova, Zlata Grigoryevskaya, Irina Petukhova, Nataliya Bagirova, Mikhail Romanov, Marina Dyachkova, Maxim Morozov, Vladimir Veselovsky, Vera Kanaeva, Dmitry Kalinin, Andrey Larin, Egor Shitikov, Ksenia Klimina

**Affiliations:** a Lopukhin Federal Research and Clinical Center of Physical-Chemical Medicine of Federal Medical Biological Agency, Moscow, Russia; b Lomonosov Moscow State University, Moscow, Russia; c Federal State Budgetary Institution «N.N. Blokhin National Medical Research Center of Oncology» оf the Ministry of Health of the Russian Federation, Moscow, Russia; d A.V. Vishnevsky National Medical Research Center of Surgery of the Russian Ministry of Healthcare, Moscow, Russia

**Keywords:** Melanoma, immune checkpoint inhibitors, gut microbiota, microbiome markers, metagenome sequencing, russian patient, bacterial predictors of immunotherapy

## Abstract

Melanoma is an aggressive malignancy with a significant risk of mortality. In recent years, treatment strategies have undergone a paradigm shift with the advent of immunotherapy, particularly immune checkpoint inhibitors (ICIs). Despite notable clinical success, a substantial proportion of patients fail to respond or eventually develop resistance to ICIs. Emerging evidence highlights the gut microbiota as a critical modulator of host immune responses and is one of the potential determinants of immunotherapy efficacy. We performed a cross-cohort analysis of gut microbiome profiles from melanoma patients treated with ICIs. The study integrated the first Russian cohort (62 patients) with six previously published international datasets, comprising a total of 490 patients across seven cohorts. In all cases, metagenomic sequencing was performed using various Illumina platforms, and raw sequencing data were processed using a unified bioinformatic pipeline. Analysis revealed 527 metagenome-assembled genomes (MAGs) significantly associated with treatment outcome: 239 with response and 288 with non-response. Notably, the species *Faecalibacterium sp900539945*, *Phocaeicola vulgatus*, *Bifidobacterium adolescentis*, *Faecalibacterium taiwanense*, and *Gemmiger qucibialis* were consistently associated with response, while *Enterobacter ludwigii* was linked to non-response. Analysis of the Russian cohort revealed both conserved and population-specific microbial signatures, highlighting the coexistence of globally shared and region-dependent microbiome features. Our results also show that species-level annotations may obscure opposing response associations within the same taxa, highlighting the need for MAGs or strain profiling. Together, this study demonstrates that cross-cohort analysis enables the identification of robust and reproducible bacterial markers of immunotherapy response, providing a foundation for microbiome-based prediction and modulation strategies in melanoma.

## 1. Introduction

Melanoma is one of the most aggressive types of human cancer, marked by a high tendency to metastasize. Over the past decade, the introduction of immune checkpoint inhibitors (ICIs) has substantially improved treatment outcomes, particularly through combination therapies targeting CTLA-4 and PD-1. These regimens have significantly increased the five-year survival rate.^1^ ^,^ ^2^ Despite the effectiveness of ICIs, stimulating the immune system can lead to autoimmune reactions.^3^ Approximately half of patients do not experience a significant reduction in tumor size following treatment, and the therapy can cause severe side effects such as dermatitis, colitis, hepatitis, thyroid dysfunction, and pneumonitis.^4-6^ This underscores the need to identify additional factors that influence treatment efficacy and could serve as predictors of therapeutic response.

Recent studies have demonstrated that the gut microbiome (GM) plays a key role in modulating the antitumor immune response. Both experimental and clinical studies have shown that the composition and functional activity of the GM can significantly influence the efficacy of immunotherapy, thereby opening new opportunities for personalized treatment strategies.^7^ ^,^ ^8^

Further support for a causal relationship between microbiota composition and treatment response comes from fecal microbiota transplantation experiments, in which fecal samples from responder patients were transferred to germ-free mice bearing melanoma tumors. This intervention was shown to enhance the antitumor effects of ICIs.^9^ ^,^ ^10^

Therefore, continued investigation into the role of the GM may provide new avenues for optimizing immunotherapy outcomes. Pre-treatment analysis of microbial composition and functional capacity could facilitate the development of targeted probiotic or microbiota-modulating interventions aimed at enhancing the efficacy of ICIs.

The beneficial effects of the GM on immunotherapy efficacy are largely mediated by metabolites they produce. Among these, short-chain fatty acids (SCFAs)—such as acetate, propionate, and butyrate—play a central immunomodulatory role.^11^ SCFAs attenuate inflammation by inhibiting nuclear factor κB (NF-κB) activation, which reduces the production of pro-inflammatory cytokines such as tumor necrosis factor *α* (TNF-*α*). At the same time, they promote the expression of anti-inflammatory cytokines, including interleukin-10 (IL-10) and transforming growth factor *β* (TGF-*β*). SCFAs also drive the differentiation of naive T cells into regulatory T cells (Tregs), thereby contributing to immune tolerance and limiting excessive immune activation.^12^ Other bacterial metabolites also have been implicated in modulating antitumor immunity. Low-molecular-weight messengers such as indoles and inosines can enhance immune responses, while trimethylamine N-oxide, produced by certain microbial species, activates CD8+ T cells and promotes tumor cell destruction.^13^ Muramyl dipeptide (MDP) and N-acetylglucosamine–MDP, derived from peptidoglycan hydrolysis by *Enterococcus* SagA hydrolases, may stimulate the NF-κB signaling pathway and exert antitumor effects.^14^ ^,^ ^15^

Multiple studies have shown that specific bacterial taxa are associated with either favorable or unfavorable clinical outcomes, underscoring the potential of the GM as a predictive biomarker of immunotherapy efficacy.^16-18^ However, findings across studies are often inconsistent, likely due to differences in patient populations, sequencing depth, and analytical methodologies. These inconsistencies highlight the need for more in-depth and standardized investigations into the relationship between microbiota composition and treatment outcomes.^19^ ^,^ ^20^

Importantly, to date, no GM studies involving melanoma patients from Russia have been published, leaving a geographic gap in the current understanding of microbiome–immunotherapy interactions. Here, we present the first metagenomic analysis of the GM in a Russian dataset of melanoma patients and perform a cross-cohort comparative analysis by integrating this dataset with six publicly available datasets. This approach enabled us to identify bacterial taxa potentially associated with immunotherapy outcomes and to determine bacteria commonly linked to treatment response across different populations.

The aim of this study was to identify GM markers associated with response to ICIs in melanoma using a unified analytical framework across multiple cohorts. We hypothesized that consistent response-associated microbial features would emerge at the strains (metagenome-assembled genomes; MAGs) level across geographically distinct populations, despite heterogeneity at the species level. In addition, we anticipated that species-level profiles may obscure opposing associations driven by strain-level heterogeneity, motivating genome-resolved analysis.

## 2. Materials and methods

### 2.1. Sample collection

**Figure 1.**
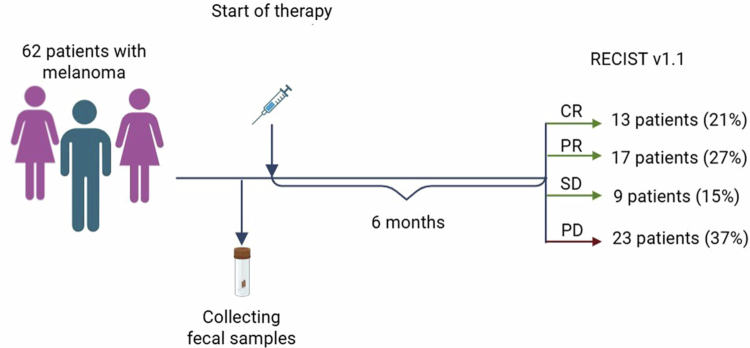
Study design and sampling scheme of the Russian cohort.

A total of 62 fecal samples were collected from adult melanoma patients receiving ICIs therapy at the Federal State Budgetary Institution «N.N. Blokhin National Medical Research Center of Oncology» оf the Ministry of Health of the Russian Federation (hereinafter referred to as N.N. Blokhin NMRCO). Samples were collected prior to treatment and grouped into two cohorts based on the year of collection: Cohort A (А_1-A_23), consisting of 23 samples collected in 2023, and Cohort B (B_24-B_69), consisting of 39 samples collected in 2024 (Figure 1). All samples were collected in sterile tubes and stored at –80 °C until use. Stool collection was performed during patient hospitalization for antitumor treatment, prior to initiating immunotherapy. The study was approved by the Ethics Committee of N.N. Blokhin NMRCO (Protocol No. 10) and the Lopukhin FRCC PCM (Protocol No. 2023/10/17), and written informed consent was obtained from all participants. Inclusion criteria required the presence of measurable disease according to the Response Evaluation Criteria in Solid Tumors (RECIST v1.1).^21^ Patients received anti-PD-1 therapy with Prolgolimab, Pembrolizumab, or Nivolumab, in accordance with the manufacturers’ recommendations. Follow-up examinations and computed tomography scans were performed after 6 months. Patients with a complete response (CR), partial response (PR) or stable disease (SD) according to RECIST criteria were classified as responders (R). Non-responders (NR) were defined as patients with progressive disease (PD). For analysis, samples from both cohorts were merged into a unified dataset.

### 2.2. DNA extraction

For Cohort A (A_1-A_23), DNA was provided by the N.N. Blokhin NMRCO. For the 39 fecal samples in Cohort B (B_24-B_69), DNA was extracted using the MagicPure® Stool and Soil Genomic DNA Kit (TransGen Biotech, China). Each sample was resuspended in 400 µL of PBS and transferred to MagNA Lyser Green Beads tubes (Roche, Switzerland), and homogenized using a MagNA Lyser (30 seconds at 7,000 rpm) (Roche, Switzerland). Following homogenization, samples were centrifuged for 1 minute at 9,000 g. The supernatant was transferred to a clean tube, and 40 µL proteinase K was added. Samples were incubated at 65 °C for 20 minutes and then transferred to a deep-well plate (Thermo Fisher Scientific, USA) containing magnetic beads from MagicPure® Stool and Soil Genomic DNA Kit (TransGen Biotech, China). Further DNA extraction was carried out on a KingFisher™ Purification System (Thermo Fisher Scientific, USA) according to the manufacturer's protocol. DNA concentration was quantified using a Qubit 4 fluorometer with the Quant-iT dsDNA BR Assay Kit (Thermo Fisher Scientific, USA).

### 2.3. Library preparation and Illumina sequencing

Library preparation was performed using 100 ng of DNA and the KAPA HyperPlus Kit (Roche, Switzerland), following the manufacturer’s protocol. Final cleanup was conducted using KAPA HyperPure Beads (Roche, Switzerland). Library size distribution and quality were assessed using a High Sensitivity DNA Chip (Agilent Technologies, USA). Quantification was performed using Quant-iT DNA Assay Kit, High Sensitivity (Thermo Fisher Scientific, USA). Libraries were pooled equimolarly and diluted to a final concentration of 750 pM. Sequencing was carried out on a NextSeq 1000 platform (Illumina, USA) using the NextSeq 1000/2000 P2 Reagents kit (200 Cycles) v3, with 2% PhiX (Illumina, USA) added as an internal control. Samples from Cohorts A and B were sequenced in two separate runs. The raw data have been deposited in the NCBI GenBank database (PRJNA1263414).

### 2.4. Bioinformatics analysis

#### 2.4.1. Datasets

In addition to the Russian cohort, 428 samples from six publicly available datasets were included in the analysis. Raw data from six available datasets were downloaded from the Sequence Read Archive using the following accession numbers ERP104610,^22^ SRP115355,^23^ SRP116709,^9^ SRP340838,^24^ ERP127050^25^ and SRP457755.^26^ All data included in the analysis were selected to meet the following criteria:

- cancer type: metastatic melanoma;
- stool samples were collected at a time point prior to the start of therapy;
- patients had received ICIs therapy, including anti-PD-1, anti-CTLA-4, or combination regimens;
- each sample was accompanied by detailed metadata that included clinical response to immune checkpoint blockade, enabling classification as R or NR;
- clinical response was assessed according to RECIST v1.1;
- raw sequencing data were available in a publicly accessible repository;
- all studies employed whole-metagenome shotgun sequencing using the different Illumina platforms (paired-end reads).

#### 2.4.2. Data preprocessing and analysis

For each sample, the quality of raw reads was assessed using FastQC v0.12.1 (https://www.bioinformatics.babraham.ac.uk/projects/fastqc/). Raw reads were trimmed using fastp v0.23.4 with the parameter “--average_qual 30”.^27^ Host-derived reads were removed by aligning the trimmed reads to the human reference genome (GRCh38) using HISAT2 v2.2.1 with the “--very-sensitive” parameter.^28^ Trimmed and filtered reads were assembled using MEGAHIT v1.2.9.^29^ Contigs shorter than 1 kb were excluded from downstream analyzes.

**Figure 2.**
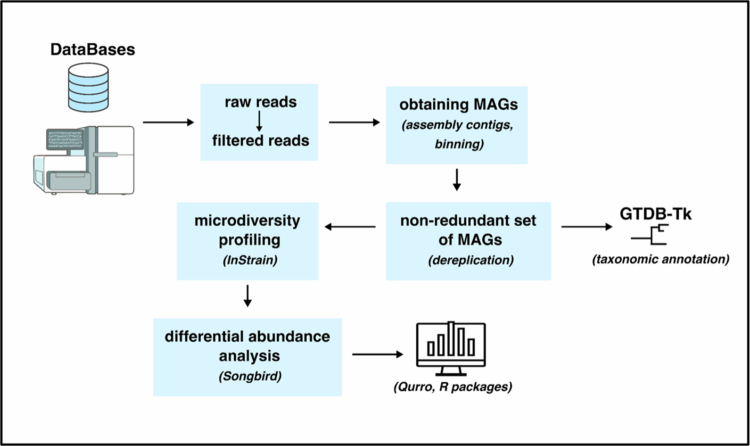
Data processing algorithm.

Metagenome-assembled genomes (MAGs) were reconstructed using three binning algorithms (MetaBAT v2.12.1,^30^ SemiBin2 v2.1.0,^31^ MaxBin v2.2.7.^32^ Binning results were integrated using DAS Tool v1.1.7.^33^ MAGs quality and completeness were assessed using CheckM v1.2.2^34^ and MAGs were dereplicated using dRep v3.4.5 (primary clustering at 90%, secondary clustering at 98% ANI).^35^ Taxonomic annotation of the resulting non-redundant set of MAGs was performed using Genome Taxonomy Database Toolkit (GTDB-Tk) v2.4.1 with database release R226.^36^ Further details on the algorithm used to generate the non-redundant MAGs set are provided in our previous publication^17^ and are available on GitHub (https://github.com/kanaevavera/Cancer_MAGs). The data processing workflow is summarized in Figure 2. Raw sequencing data from all seven datasets, including our own dataset, were processed through a unified bioinformatics pipeline to minimize technical variability.

Population microdiversity metrics were calculated using inStrain v1.9.0^37^ against the non-redundant MAG set as reference. Matrices of MAGs relative abundances for each sample were computed using a custom Python script from inStrain output. Genomes with coverage width less than 0.5 were excluded from further analysis. The resulting abundance matrices were used as input for Songbird v1.0.4^38^ (Figure 2). Songbird was run with the following parameters: “--*p*-epochs 10000 --*p*-differential-prior 0.5 --*p*-min-sample-count 1 --*p*-min-feature-count 0 --*p*-summary-interval 1”. MAGs with differential values >0.3 or <−0.3, corresponding to an approximately two-fold change in representation, were considered most strongly associated with the R or NR group. Rank plots and log-ratios were visualized using Qurro v0.8.0.^39^ Statistical analyzes were performed in R v4.2.1. The Wilcoxon rank-sum test with Bonferroni adjustment was used to conduct paired comparisons among the groups R and NR and to assess the statistical significance of the log-ratios presented in the box plots. Box plots were generated using the R packages “ggplot2”, “rstatix” and “ggsignif”. To visualize the species associated with R and NR groups, faceted barplots were generated using Python (matplotlib, pandas), separately for each dataset. The “pheatmap” R package was used to build a heatmap representing overlaps between datasets.

## 3. Results

### 3.1. Baseline clinical characteristics and response outcomes in Russian patients

A total of 62 individuals with histologically confirmed cutaneous melanoma of either regional lymph node metastasis or local recurrence with measurable lesions were included in the study. All participants received anti-PD-1 therapy. Patients had no evidence of intestinal infection and had not received antibiotic therapy within 28 d prior to inclusion. The majority of participants were female (*n* = 38; 61.3%) with a median age of 58 y (range: 34–87 y). Based on RECIST v1.1 criteria, 39 patients (62.9%) were classified as R: 13 patients (20.9%) as CR, 17 (27.4%) as PR and 9 (14.5%) as SD. The remaining 23 patients (37.1%) experienced PD and were categorized as NR. Most patients (*n* = 48; 77.4%) had stage IIIB/C/D disease or the equivalent, while 14 patients (22.6%) had oligometastatic, resectable stage lV melanoma with measurable foci. Among the responders, 33 responders (84.6%) had an Eastern Cooperative Oncology Group (ECOG) performance status of 0. Of the 62 patients 34 (54.8%) harbored a BRAF mutation. Among these, 30 patients (88.2%) carried a V600 E/K BRAF mutation, while 4 patients (11.8%) had a non-V600 E/K BRAF mutation. The majority of responders (*n* = 22; 56.4%) had a V600E/K BRAF mutation. Elevated lactate dehydrogenase (LDH) levels were in 7 responders (17.9%). Detailed cohort characteristics are presented in Supplementary Table S1.

### 3.2. Responders and non-responders harbor distinct gut microbiome communities

To investigate differences in GM composition between therapy responders and non-responders, we analyzed seven publicly available metagenomic datasets, comprising a total of 490 fecal samples (286 R and 204 NR; Table 1). The selection of studies was based on specific criteria described in the Materials and Methods section. Detailed information for each sample is provided in Supplementary Table S2.

**Table 1.** Summary of the datasets included in the analysis.

No.	Dataset	Amount	Immunotherapy	Response (R/NR)	Number of MAGs (R/NR)	Number of species (R/NR)	Instrument Illumina	Read length, bp	Country
1	NN_Blokhin_NMRCO	62	anti-PD-1	39/23	347/399	116/193	NextSeq 1000	2*100	Russia: Moscow
2	Frankel et al.^23^	39	anti-PD-1 and/or anti-CTLA-4	24/15	314/280	124/169	HiSeq 2000	2*100	USA: Dallas, Texas
3	Matson et al.^9^	39	anti-PD-1 or anti-CTLA-4	15/24	166/122	95/87	NextSeq 500	2*150	USA: Chicago, Illinois
4	Gopalakrishnan et al.^22^	22	anti-PD-1	11/11	136/67	70/50	HiSeq 2000	2*100	USA: Houston, Texas
5	Spencer et al.^24^	134	anti-PD-1 and/or anti-CTLA-4	88/46	370/417	176/236	HiSeq 2000 HiSeq Х	2*100 2*150	USA: Houston, Texas
6	Lee et al.^25^	165	anti-PD-1 and/or anti-CTLA-4	94/71	573/652	226/312	NovaSeq 6000	2*150	Europe: Spain, United Kingdom, Netherlands
7	Tsakmaklis et al.^26^	29	anti-PD-1 and/or anti-CTLA-4	15/14	218/233	122/158	NovaSeq 6000	2*150	Germany: Cologne
	**Total**	**490**		**286/204**	**2124/2170**	**929/1205**			

**Figure 3.**
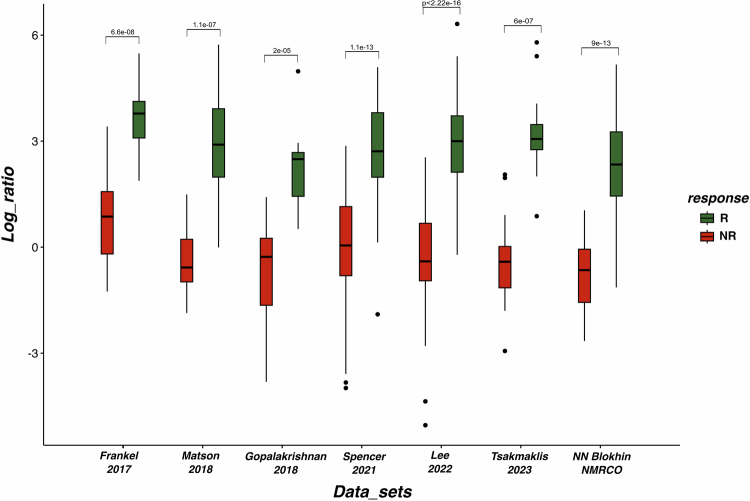
Box plot showing the log-ratio of selected MAGs between R (green) and NR (red) across seven datasets. Differences between groups (R vs. NR) were assessed using the Wilcoxon rank-sum test with Bonferroni correction, all comparisons are statistically significant (*p* < 0.0001).

To test whether response to immunotherapy correlates with differences in GM composition, we compared the relative abundance of MAGs between R and NR patients across all seven datasets. We began by analyzing the rank plots, which directly visualize top‑changing MAGs and are presented in Supplementary Figure S1. All rank plots of MAGs differentials display a characteristic asymmetry, consistently showing a longer tail in NR compared to R. This extended tail indicates a larger number of MAGs with relatively low abundance values, suggesting broader diversity of impactful, albeit less dominant, microbial features (MAGs) associated with non‑response. Focusing only on the MAGs that changed most between R and NR (highlighted in red and blue in the rank plots—Figure S1), we constructed box plots to assess whether the differences between the two groups were statistically significant. Across all datasets, the log ratios of these microbial features (MAGs) differed significantly between R and NR (Figure 3).

**Figure 4.**
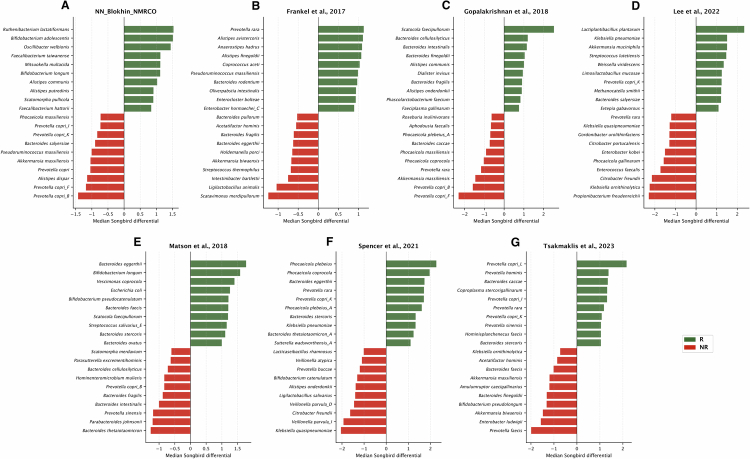
Species-level Songbird differentials across seven metagenomic datasets (panels A–G). For each dataset, the top 10 species with the highest positive (R-associated, green) and negative (NR-associated, red) median differentials are shown. Only species with formally assigned binomial names are shown.

Having established that R and NR groups harbor significantly different microbial communities across all seven datasets (Figure 3), we next examined which specific bacterial species drive these differences. To assess taxonomic differences between R and NR, we analyzed annotated MAGs significantly associated with each group (differential values exceeding |d| > 0.3) per dataset (Figure 4). The number of detected MAGs varied across datasets, reflecting differences in sequencing depth: datasets with higher read counts yielded greater MAG diversity and broader microbiota coverage (Table 1). Despite this variability, several patterns recurred across datasets: species associated with R mainly belonged to the genera *Faecalibacterium*, *Alistipes*, *Bifidobacterium*, and *Phocaeicola*, while NR-associated species were characterized by members of *Bacteroides*, *Prevotella*, and opportunistic taxa such as *Klebsiella*, *Citrobacter,* and *Enterobacter*. The full list of Songbird differentials and taxonomy for each MAG per dataset is provided in Supplementary Table S3.

### 3.3. Species-level differences: unique taxa in the Russian dataset

In this study, we reprocessed all datasets using a unified bioinformatic framework and additionally evaluated the proportion of species that were unique to each cohort within the responder and non-responder groups. A substantial proportion of bacterial species was unique to each dataset. Among R, the proportion of unique species ranged from 14.7% to 42.9%, while in NR it ranged from 19.5% to 37.5%. Detailed lists of bacterial species unique to each dataset are provided in Supplementary Table S4.

In the Russian cohort, 41 unique bacterial species were associated with R, and 52 unique species were linked to NR. According to the literature, we highlight several representative taxa from each group that are of particular interest due to their potential impact on the host immune system and metabolic processes. Among the species associated with a positive response were *Vescimonas fastidiosa*, *Megasphaera elsdenii*, *Gallintestinimicrobium propionicum*, *Eggerthella lenta*, *Catenibacterium mitsuokai*, and *Mitsuokella multacida*. In contrast, unique species linked to non-response included *Prevotella copri_K* and *Butyricimonas virosa*. It should be noted that other bacterial taxa were also identified; however, many of them remain poorly characterized, likely due to their limited prevalence across populations.

### 3.4. Limitations of species-level analysis

Through comparative analysis of the microbial profiles, we identified a limited set of bacterial species that were consistently present in both responders and non‑responders. Comprehensive dataset‑stratified inventories of these shared species are provided in Supplementary Table S5. Specifically, in the Russian cohort, 22 such species were detected in both groups. However, the strain-level profiles within these species differed between the groups. Examples with valid binomial names include *Ruminococcoides intestinale*, *Faecalibacterium longum, Faecalibacterium prausnitzii, Ellagibacter isourolithinifaciens, Agathobacter rectalis, Agathobaculum butyriciproducens, Akkermansia muciniphila, Vescimonas cirricatena, Vescimonas coprocola* and *Escherichia coli* (genome‑based placeholder taxa—e.g., GTDB labels such as CAG‑103 sp900543625—are provided in Supplementary Table S5).

We focused on the genus *Faecalibacterium* as an illustrative case study. Previous studies have repeatedly identified this genus as one of the most robust microbiome-associated predictors of favorable response to ICIs in melanoma.^22^ ^,^ ^24^ Importantly, our cross-cohort analysis indicates that representatives of this genus are detected in both R and NR across all datasets (Supplementary Table S5). Therefore, we performed a more detailed genome-resolved analysis to clarify the role of *Faecalibacterium* in the context of ICI response.

In the Gopalakrishnan et al. dataset,^22^ *Faecalibacterium wellingii*, *Faecalibacterium taiwanense*, *Faecalibacterium duncaniae*, *Faecalibacterium sp900539945* were associated with responders, whereas *F. prausnitzii* appeared in both groups. According to updated data in Spencer et al. dataset,^24^ the species *F. sp900539945*, *Faecalibacterium sp934514285*, *Faecalibacterium butyricigenerans*, *F. taiwanense*, *Faecalibacterium tardum*, *Faecalibacterium sp900540455*, *F. wellingii* were associated with responders, and *Faecalibacterium intestinale* was associated with non-responders. *F. longum*, *F. prausnitzii*, *F. prausnitzii_E*, and *F. duncaniae* were present in both groups. In the first analyzed Russian cohort, this species-specific pattern persists: five *Faecalibacterium* species—*Faecalibacterium hattorii*, *F. taiwanense*, *F. prausnitzii_I*, *F. tardum*, and *F. sp900539945*—were associated with R, while *F. prausnitzii_E* and *F. prausnitzii_F* were linked to NR.

In the course of analyzing all datasets, it was discovered that 14 *Faecalibacterium* species were associated with R and 12 species with NR groups. Specifically, *F. tardum*, *F. sp900539945*, *F. taiwanense*, and *F. sp900540455* were identified exclusively in R and may be considered beneficial species linked to favorable immunotherapy outcomes. In contrast, *F. prausnitzii_F* and *Faecalibacterium faecigallinarum* were observed only in NR. Among these, only six species were unique and did not overlap. This pattern suggests the presence of multiple strains that may exert distinct effects on treatment outcomes, in line with emerging evidence that strain-level variation can significantly influence host-microbe interactions.^40^ ^,^ ^41^

### 3.5. Cross-cohort MAGs analysis identifies robust microbial markers of immunotherapy response

Given that a single bacterial species may encompass strains with opposing effects on immunotherapy outcomes, identifying robust microbial markers of treatment efficacy requires finer taxonomic resolution, for example at the MAGs level. To address this, we performed a cross-cohort analysis of MAGs, applying a series of filtering criteria. Only MAGs that were detected in at least two independent datasets and showed a consistent association with response across all datasets in which they were present were included (i.e., they were associated exclusively with either R or NR in all relevant datasets). For this analysis, we used MAGs previously selected based on differential values: - 0.3 > response > 0.3.

Across all datasets, we identified 527 MAGs that significantly linked to immunotherapy outcomes. Among these, 288 MAGs were associated with NR, while 239 MAGs—with R (Supplementary Table S6—complete table of cross-cohort microbial markers). Notably, MAGs linked to a favorable immunotherapy response were frequently shared across five, six, or even all seven datasets. In contrast, MAGs associated with NR generally overlapped across fewer datasets, typically two to three datasets.

**Figure 5.**
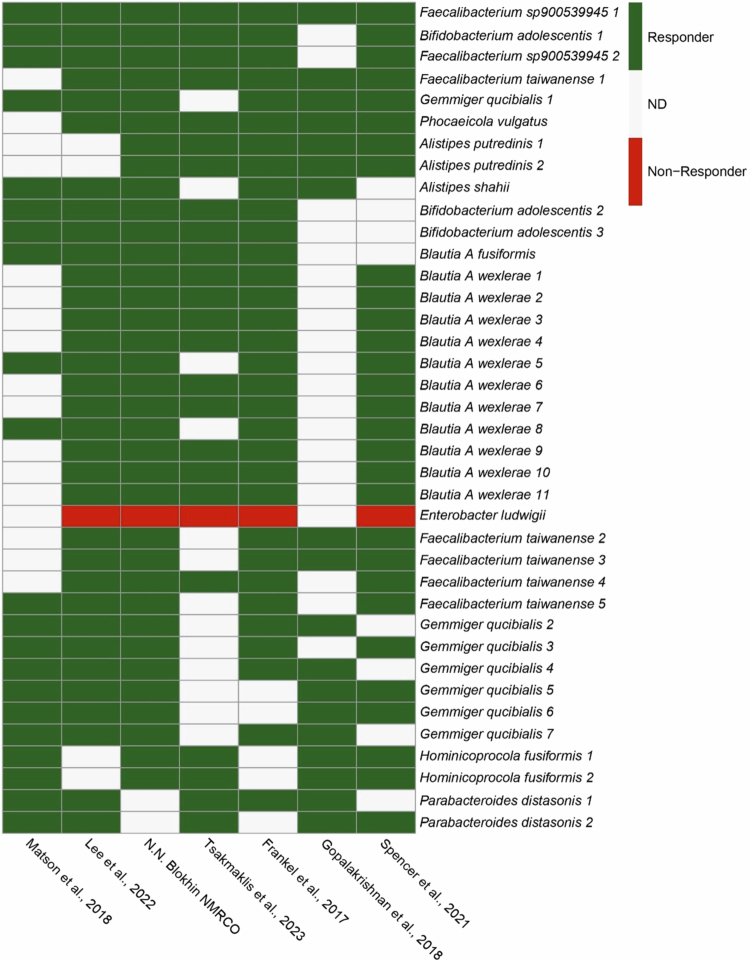
Overlap of MAGs associated with R and NR to immunotherapy across five or more datasets. Different MAGs (strains) assigned to the same species marked in the figure by unique numeric labels. ND (not detected) indicates that MAG is not associated with the outcome of immunotherapy in the given dataset. Data for other MAGs associated with immunotherapy response are presented in Supplementary Figure S2.

We found that a MAG belonging to bacterial species *F.* sp900539945 was consistently identified across all seven studies and was associated with a positive therapeutic response. Five other MAGs were shared across six studies, all of which were associated with responders: *Phocaeicola vulgatus*, *Bifidobacterium adolescentis*, *F. taiwanense*, *F.* sp900539945, and *Gemmiger qucibialis*. Thirty-two MAGs were shared across five of the analyzed datasets (Figure 5). While the majority of these were associated with a positive response to immunotherapy, the exception was *Enterobacter ludwigii*, which was linked to non-responders.

An overlap of 61 MAGs was observed across four datasets, with 51—associated with a R and 10—associated with NR. In three datasets, 111 MAGs overlapped, including 53 associated with R and 58 associated with NR. Finally, 317 MAGs overlapped between two studies, of which 219 were linked to NR and 98 to R. All discussed MAGs, corresponding to bacterial species are presented in Supplementary Figure S2.

## 4. Discussion

Numerous studies have demonstrated that the composition of the GM differs between patients who respond to ICIs therapy and those who do not.^42-49^ These findings underscore the GM’s critical role as a key factor influencing the success of immunotherapy. However, it is important to note that the approaches to data analysis and the list of identified markers varied significantly across different studies. This heterogeneity highlights the need for standardized cross-cohort data processing and for the identification of robust, reproducible microbiome features associated with treatment response.

In the present study, we performed a MAG-resolved cross-cohort analysis integrating the first Russian melanoma dataset with six previously published international cohorts. By processing all datasets from raw sequencing reads using a unified analytical pipeline, we were able to compare microbial features across diverse cohorts. Although DNA extraction protocols differed between cohorts, which represents a limitation of this work,^50^ all datasets were generated using Illumina-based whole-metagenome shotgun sequencing and underwent identical downstream processing, thereby reducing technical variability at the sequencing and analysis levels.

Our analysis reproduced key associations reported in earlier research and provided new insights into the microbiota—immunotherapy relationship. Notably, species associated with R showed greater overlap across datasets compared to NR-associated species. We interpret this as evidence that in responders the presence of more stable microbial communities with a balanced/dominant set of key bacterial taxa is observed, as well as functional stability and metabolic homeostasis—provided by the established community. Thus, patients enter immunotherapy harboring this stable microbiota. By contrast, NR exhibit greater heterogeneity—higher between-individual variability—consistent with the “Anna Karenina principle” for animal microbiomes.^18^ ^,^ ^19^ ^,^ ^51^ Furthermore, we believe that this heterogeneity, among other things, manifests as a larger number of weak yet significant MAGs in NR and the characteristic long tail on differential rank plots (Supplementary Figure S1). Despite the observed species-level consistency in microbial taxa associated with response across datasets (Figure 4), we also identified unique species that were specific to individual populations. The presence and abundance of these taxa may be influenced by ecological factors such as geographic region, diet, and lifestyle.^52^

A common feature of bacteria associated with a positive response is their ability to produce metabolites that beneficially affect the host—primarily SCFAs and nucleotides. This pattern is also evident among the unique bacteria identified in the Russian dataset. For example, *M. multacida* is capable of metabolizing phytates derived from plant-based foods into SCFAs.^53^ *M. elsdenii* exhibits a broad spectrum of metabolic activity: it produces butyrate, acetate, formate, caproate, as well as vitamins and essential amino acids.^54^ Moreover, it synthesizes pentanoate—a metabolite shown to affect CD4+ T cell function by inhibiting histone deacetylases and altering their epigenetic status.^55^

*G. propionicum* is also presumed to synthesize butyrate, although its metabolic pathways require further investigation.^56^ Of particular interest is *E. lenta*, whose increased abundance in R correlates with CD8+ T cell activation and enhanced cytotoxic response. These effects have been reported in previous experimental studies and may provide associations observed in our analysis.^57^ *E. lenta* synthesizes and releases nucleotides and nucleotide metabolism intermediates, as well as molecules such as indole-3-acetate and inosine, which are capable of modulating immune signaling pathways.^58^

It is important to note that the bacteria described may exert beneficial effects in other populations as well; however, they appear more frequently in the Russian dataset, which is why they are identified as significantly associated with R. For instance, *C. mitsuokai*, a known SCFA producer, has been reported to occur more commonly among individuals living in rural areas.^59^ Its presence among the unique bacterial species in the Russian dataset may therefore reflect regional prevalence, despite being detected in other populations as well.^52^

Identifying common traits among bacteria associated with NR is more challenging. However, among the unique taxa characteristic of NR in the Russian dataset, *B. virosa* was identified. This species possesses potential pathogenic properties, particularly under conditions of weakened immune responses caused by the underlying disease.^60^ Another example is *P. copri*, which has been linked to the development of autoimmune diseases. These bacteria can initiate both local and systemic immune responses, leading to impaired intestinal barrier function—especially in the context of concomitant chemotherapy.^61^ ^,^ ^62^ Although *P. copri* is sometimes found in R patients,^63^ it may also be associated with therapy failure.^42^ This highlights the importance of considering strain-level variability when interpreting the immunomodulatory role.

Apparently, even very closely related bacterial species can drive differences in therapeutic response. One of the clearest examples of such conserved yet complex microbial associations is the genus *Faecalibacterium*. Growing evidence suggests substantial genomic and functional diversity within its members.^64^ Representatives of this bacterial genus have been repeatedly associated with positive outcomes of immunotherapy^65^ widely postulated its role in butyrate production and gut barrier support.^41^ ^,^ ^66^ Our findings indicate a more nuanced relationship between this genus and treatment outcome than previously recognized. Therapeutic efficacy varies not only among *Faecalibacterium* species but also among strains within a single species. Therefore, accurate interpretation of the results requires resolution not only at the species level but also at the strain level. Indeed, *F. prausnitzii* strains differ markedly in gene content and immunomodulatory activity,^67-70^ supporting the notion of genetically distinct groups within a single taxonomic label. By applying a uniform analytical framework across all datasets, we were able to reveal this intra-genus heterogeneity and demonstrate that even microbes traditionally viewed as beneficial may show divergent associations with ICIs outcomes—likely due to underlying strain-level differences.

Our analysis identified several MAGs associated with a positive response in five or more of the seven datasets. According to the literature, the identified GMs markers not only correlate with the response to immunotherapy in other studies, but also hold the potential to positively influence human anti-tumor immune responses. *Faecalibacterium* sp. 900539945 was frequently observed in responders’ metagenomes across all analyzed datasets, consistent with other studies linking this species to favorable immunotherapy outcomes.^19^ The *Bifidobacterium* genus is of particular interest in melanoma studies; for example, molecular mimicry between melanoma and *Bifidobacterium*-derived epitopes may promote cross-reactive T cell activation.^71^ *B. adolescentis* has been shown to enhance the anti-tumor immune response to ICIs in murine models.^72^ In humans, *B. adolescentis* presence correlates with positive outcomes in metastatic melanoma patients.^9^ *Bifidobacteria*, in general, contribute to host health through the synthesis of SCFAs, such as butyrate, which possess anti-inflammatory and anti-carcinogenic properties.^73^ Similarly, certain strains of *Alistipes shahii* are known to produce butyrate, reduce intestinal inflammation, and alleviate symptoms of Inflammatory Bowel Disease (IBD).^74^ *Phocaeicola vulgatus*, associated with a positive response in the six datasets we analyzed, may exert favorable effects on health, including the amelioration of colitis. However, the impact of *P. vulgatus* is recognized as strain-specific: some strains induce inflammation via activation of TNF-*α* production, while others reduce the pro-inflammatory response through the SCFAs and capsular polysaccharides production.^75^ ^,^ ^76^

Bacterial taxa associated with responders tended to overlap across a larger number of datasets compared to those associated with non-responders. While negative microbial markers demonstrated less consistency across studies, one species—*E. ludwigii*—was associated with non‑response in five studies. This species is noteworthy for its potential role in the development of opportunistic infections, which primarily affect immunocompromised individuals.^77^ It is plausible that the observed negative association stems not from the bacterium's direct impact, but rather a reflection of the presence of nosocomial infections, which may indicate a compromised immune system in these individuals. Alternatively, proliferation of *E. ludwigii* in an immunocompromised host may further impair the immune response against malignancies such as melanoma. The absence of this pathogenic species in patients who successfully respond to treatment may also result from specific bacteriophages.

Thus, further investigation of the phage component of the GM along with the bacteria is critically important. The identification and characterization of bacteriophages capable of eliminating pathogenic bacteria opens a promising path toward developing new microbiota-correcting strategies, which can significantly increase the percentage of patients successfully responding to therapy.

## 5. Conclusion

The inclusion of the Russian cohort fills an important geographic gap in microbiome–immunotherapy research, which has thus far been dominated by datasets from Western Europe and North America, and enables evaluation of microbiome features across culturally and environmentally distinct populations, such as dietary habits, lifestyle, healthcare practices, and antibiotic use. At the same time, our analysis revealed unique bacterial markers in each dataset, demonstrating the coexistence of globally conserved microbiome signatures and population‑specific patterns.

Within this framework, processing all datasets using a unified analytical pipeline enabled the identification of 527 MAG-resolved microbial units consistently associated with treatment outcome across independent studies. Notably, several microbial markers of response were highly conserved across cohorts. *Faecalibacterium* sp.900539945 was reproducibly associated with favorable response in all seven datasets, while additional taxa, including *B. adolescentis*, *P. vulgatus*, *F. taiwanense*, and *G. qucibialis*, were shared across at least five cohorts. In contrast, microbial features associated with non-response were more heterogeneous and showed limited cross-cohort overlap, with *E. ludwigii* representing a rare example of a consistently non-response-associated taxon.

Our cross‑cohort analysis has identified stable microbiome features associated with ICI response, yet several translational steps are still required. First, longitudinal studies with serial sampling are needed to map the temporal dynamics of the microbiota during treatment and to determine whether microbial shifts precede or follow clinical changes. Second, the conserved response‑associated taxa uncovered here (e.g., *F. sp.900539945*, *B. adolescentis*) offer a rationale for designing microbiome‑modulating strategies—such as probiotic supplementation—as potential adjuvants to immunotherapy. In this way, the present findings lay both the evidence base and the conceptual framework for future interventional studies aiming to improve melanoma immunotherapy outcomes via microbiome manipulation.

## Supplementary Material

Supplementary_legends

Supplementary Material Supplementary Material

Table S3_after comments.xlsx

Table S5_after_comments.xlsx Table S5_after_comments.xlsx

Table S4_after comments.xlsx Table S4_after comments.xlsx

Table_S6_after comments.xlsx Table_S6_after comments.xlsx

Table S2_after comments.xlsx Table S2_after comments.xlsx

Fig S2_after comments.pdf Fig S2_after comments.pdf

Table S1_after comments.docx Table S1_after comments.docx

Fig S1_after comments.png Fig S1_after comments.png

## Data Availability

The raw data obtained using Illumina sequencing have been deposited in the NCBI GenBank database: BioProject accession PRJNA1263414. Raw data from six available datasets were downloaded at the Sequence Read Archive using the following accession numbers ERP104610, SRP115355, SRP116709, SRP340838, ERP127050 and SRP457755.
