# Supplementary material for: Gut microbial markers of immunotherapy response in melanoma: a cross-cohort analysis including the first Russian dataset
Source: Gut Microbes. 2026 Jun 15;18(1):2681788. doi: 10.1080/19490976.2026.2681788 (PMC13274128; doi:10.1080/19490976.2026.2681788)
Supplement: Supplementary_legends [file KGMI_A_2681788_SM8818.docx]

**The supplementary materials are available via the following link:** <https://drive.google.com/drive/folders/19022nAZ8E1OZm6DzeQIhYaIg5iV-O0ws?usp=sharing>

**Supplementary legends**

**Figure S1.** Figure shows rank plots (panels A-G) for the seven independent analyzed datasets included in our cross-cohort analysis. MAGs ranks estimated from Songbird (multinomial regression for compositional data). The y-axis represents the log-fold change, and the x-axis numerically orders the ranks of each MAG in the analysis. MAGs ranked by association with immunotherapy outcome. Differentials with a coefficient > |0.3| are highlighted (R: red, NR: blue).

**Figure S2.** Overlap of MAGs associated with R and NR to immunotherapy across four or less datasets. Different MAGs (strains) assigned to the same species marked in the figure by unique numeric labels. ND (not detected) indicates that MAG is not associated with the outcome of immunotherapy in the given dataset.

**Table S1**. Baseline patient characteristics

**Table S2.** Sequencing and sample metadata for all analyzed samples.

**Table S3.** Species significantly associated with R and NR in each dataset.

**Table S4.** Unique bacterial species in each dataset.

**Table S5.** Bacterial species detected in both R and NR groups in each dataset.

**Table S6.** Cross-cohort bacterial markers associated with response and non-response to ICI therapy.
