# Supplementary figures and images for: Gut microbial markers of immunotherapy response in melanoma: a cross-cohort analysis including the first Russian dataset
Source: Gut Microbes. 2026 Jun 15;18(1):2681788. doi: 10.1080/19490976.2026.2681788 (PMC13274128; doi:10.1080/19490976.2026.2681788)

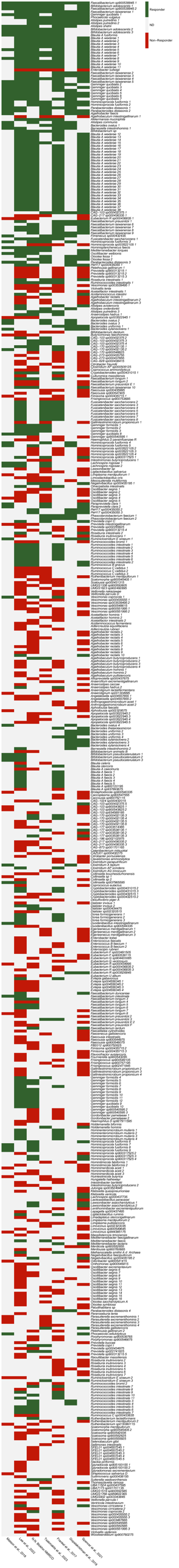

Supplement: Fig S2_after comments.pdf [file KGMI_A_2681788_SM7189.pdf]

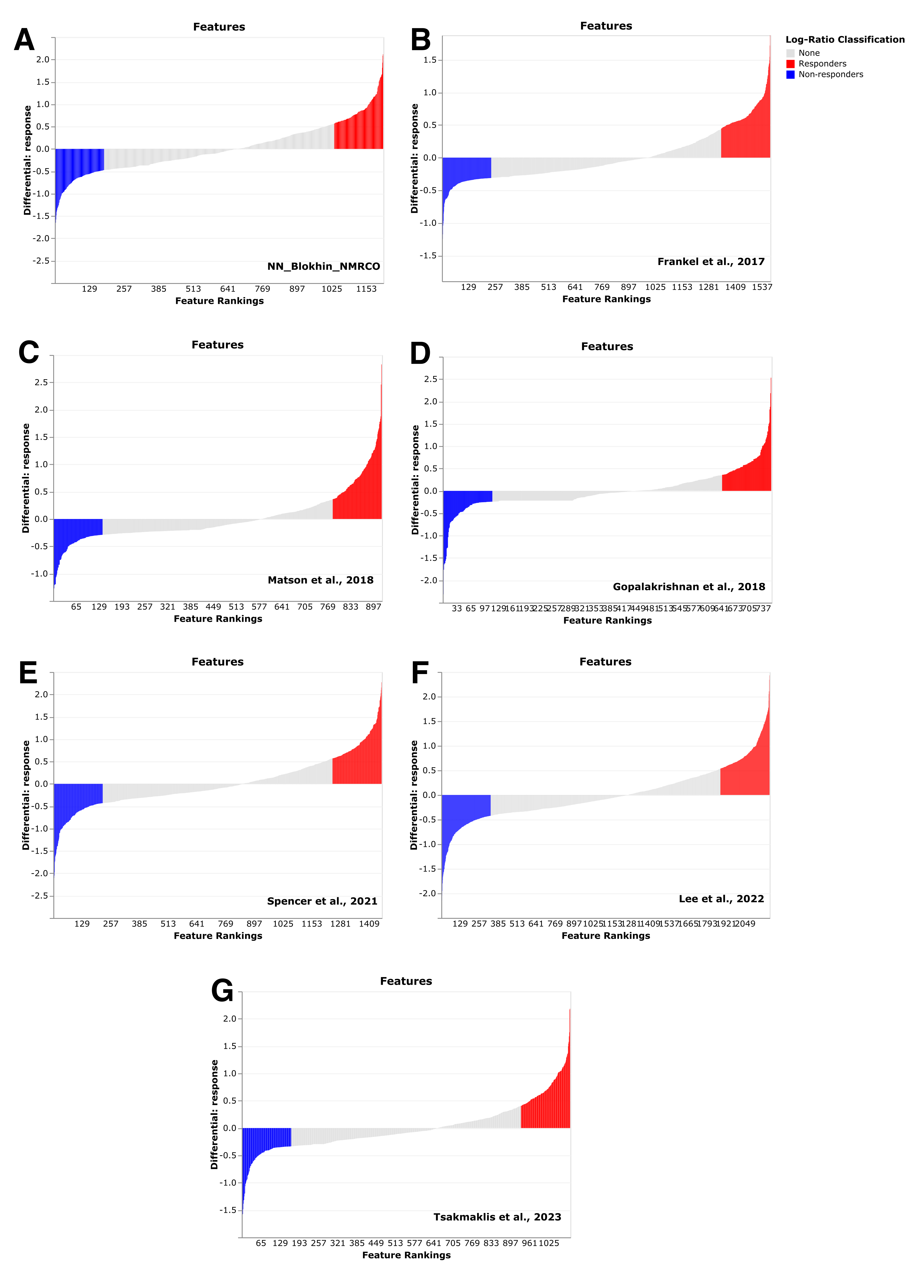

Supplement: Fig S1_after comments.png [file KGMI_A_2681788_SM7187.png]
