# Supplementary material for: Gut microbial markers of immunotherapy response in melanoma: a cross-cohort analysis including the first Russian dataset
Source: Gut Microbes. 2026 Jun 15;18(1):2681788. doi: 10.1080/19490976.2026.2681788 (PMC13274128; doi:10.1080/19490976.2026.2681788)
Supplement: Table S1_after comments.docx [file KGMI_A_2681788_SM7188.docx]

**Table S1.** Baseline patient characteristics

| **Characteristics (n = 62)** | **Cohort A (n = 23)** | | **Cohort B (n = 39)** | |
| --- | --- | --- | --- | --- |
|  | **NR (n = 12)** | **R (n = 11)** | **NR (n = 11)** | **R (n = 28)** |
| **Mean age, years, median**  **(min-max)** | 56,5 (45-85) | 63 (41-78) | 62 (40-87) | 55 (34-82) |
| **Gender, male (%)** | 7 (58%) | 4 (33%) | 3 (27%) | 10 (36%) |
| **Medicines,%** | | | | |
| Prolgolimab | 95,7 | | 69,2 | |
| Nivolumab | - | | 2,6 | |
| Pembrolizumab | 4,3 | | 28,2 | |
| **ECOG** | | | | |
| **0** | 9 (75%) | 10 (91%) | 7 (64%) | 23 (82%) |
| **1** | 3 (25%) | 1 (9%) | 4 (36%) | 5 (18%) |
| **Stage of the disease** | | | | |
| **IIIB** | 2 (17%) | 2 (18%) | 4 (36%) | 3 (11%) |
| **IIIC** | 5 (41%) | 7 (64%) | 4 (36%) | 16 (57%) |
| **IIID** | 2 (17%) | 1 (9%) | 1 (9%) | 1 (4%) |
| **IV** | 3 (25%) | 1 (9%) | 2 (18%) | 8 (29%) |
| **LDH level** | | | | |
| **Normal**  **(< 250 U/l)** | 9 (75%) | 11 (100%) | 8 (73%) | 21(75%) |
| **Elevated**  **(> 250 U/l)** | 3 (25%) | 0 | 3 (27%) | 7 (25%) |
| **BMI, median (min-max)** | 26.3  (22.8-30.7) | 25.15  (21.9 - 35.2) | 28.1  (23.1- 44.9) | 29.4  (21.3 - 43.2) |
| **BRAF mut (%)** | 5 (42%) | 7 (64%) | 3 (27%) | 15 (55%) |
| **BRAF WT (%)** | 5 (42%) | 4 (36%) | 8 (73%) | 11 (39%) |
| **BRAF other** | 2 (17%) | 0 | 0 | 2 (7%) |
